# Expression of Rho GTPases family in melanoma cells and its influence on cytoskeleton and migration

**DOI:** 10.18632/oncotarget.15618

**Published:** 2017-02-22

**Authors:** Si-Jian Wen, Wei Zhang, Na-Na Ni, Qiong Wu, Xiao-Po Wang, You-Kun Lin, Jian-Fang Sun

**Affiliations:** ^1^ Department of Dermatology and Venerology, First Affiliated Hospital of Guangxi Medical University, Nanning 530021, China; ^2^ Department of Pathology, Institute of Dermatology, Chinese Academy of Medical Sciences and Peking Union Medical College, Nanjing 210042, China; ^3^ Jiangsu Key Laboratory of Molecular Biology for Skin Diseases and STIs, Nanjing 210042, China

**Keywords:** Rho GTPase, melanoma, cytoskeleton, cell migration

## Abstract

Rho GTPases family members influenced the filopodia, lamellipodia, stress fiber and adhesion plaque of melanoma cells through regulating cytoskeleton recombination. The role of Rho GTPases family in the migration and invasion of melanoma and its molecular mechanism were explored. The morphological difference between three types of melanoma cells (M14, A375 and MV3) and human melanocyte (MC) was observed by the Hoffman microscope. Cells were stained by phalloidin labeled by rhodamine. The differences between 4 types of cells in filopodia, lamellipodia, stress fiber and adhesion plaque (microfilament is the main constituent) were observed under the super-high resolution microscope. The migration ability of 4 types of cells was detected by Transwell migration assay. QPCR was used to detect the mRNA transcription level of Rho GTPases family. WB was adopted to detect the expression of RhoD and DIAPH2 proteins. There were significant differences in filopodia, lamellipodia, stress fiber and adhesion plaque between MC and 3 types of melanoma cells (M14, A375 and MV3). MC did not have stress fiber or adhesion plaque, while M14, A375 and MV3 had stress fiber and adhesion plaque. All 4 types of cells had thin and short filopodia. MV3 had fewer but thicker stress fibers than the latter two. Transwell migration test indicated the followings: M14 and A375 had a similar high migration rate; the migration rate of MV3 was slightly low; MC did not have the ability of transmembrane migration. QPCR results of Rho GTPases family in 4 types of cells showed the change corresponding to immunofluorescence. WB results showed that RhoD was barely expressed in M14, A375 or MV3. DIAPH2, the downstream effector molecule of RhoD, had the corresponding change. Rho GTPases influences the migration and invasion of melanoma cells through regulating filopodia, lamellipodia, stress fiber and adhesion plaque (microfilament is the main constituent).

## INTRODUCTION

The invasion and metastasis of melanoma is the main reason for patient death, and badly threatens patients’ life safety [1]. The invasion and metastasis of tumor include multiple processes, of which cell movement is a must [2, 3]. Factors influencing cell movement will inevitably influence the migration and invasion of the tumor [4]. Strenuous recombination of cytoskeleton can influence the cell movement. Rho GTPases family is a key regulatory factor of cytoskeleton recombination.

In recent years, abnormal expression of Rho GTPase has been found in many malignancies. The cell movement mediated by Rho GTPase plays an extensive and important role in the invasion and metastasis, malignant transformation, regulation and proliferation of malignant cells as well as tumor angiogenesis [5]. The relationship between Rho GTPase family and melanoma has been reported occasionally. However, the expression of the whole family in melanoma cell lines, the relationship between Rho GTPase family and cytoskeleton, cell migration and invasion ability, and possible mechanisms remains unclear.

Therefore, the present study observed the differences in morphology and cytoskeleton between three types of melanoma cells (M14, A375 and MV3) and MC, investigated the movement and migration ability of 4 types of cells by Transwell chamber, and studied the transcription of Rho GTPases family and the expression of certain proteins in its downstream by QPCR and Western blot, respectively, so as to elaborate the role of Rho GTPases in the invasion and migration of melanoma cells and its molecular mechanism.

## RESULTS

### Morphology of MC and melanoma cells

**Figure 1:**
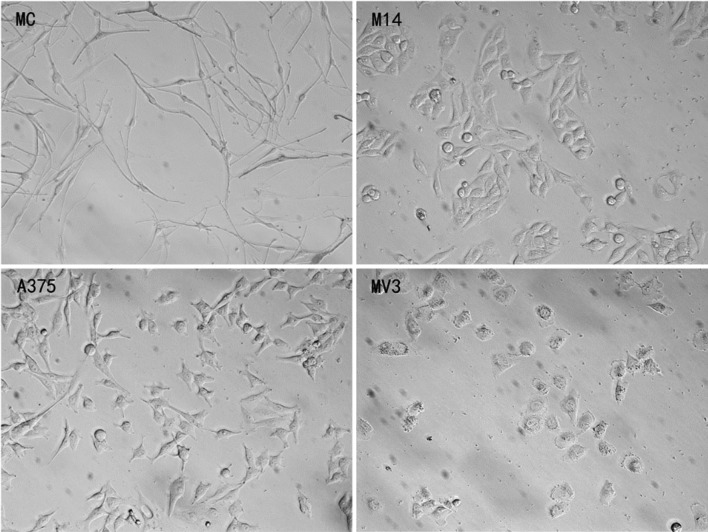
Morphology of 4 types of cells observed under the Hoffman microscope

As shown in Figure 1, MC, M14, A375 and MV3 had different morphology. MC had small body, and generally had 2-3 thin and symmetrical lamellipodia. A375 and M14 were spindle in shape and had wide lamellipodia at both ends. MV3 cells had fried egg shaped appearance.

### Similarities and differences of cytoskeleton were observed after immunofluorescence staining

**Figure 2:**
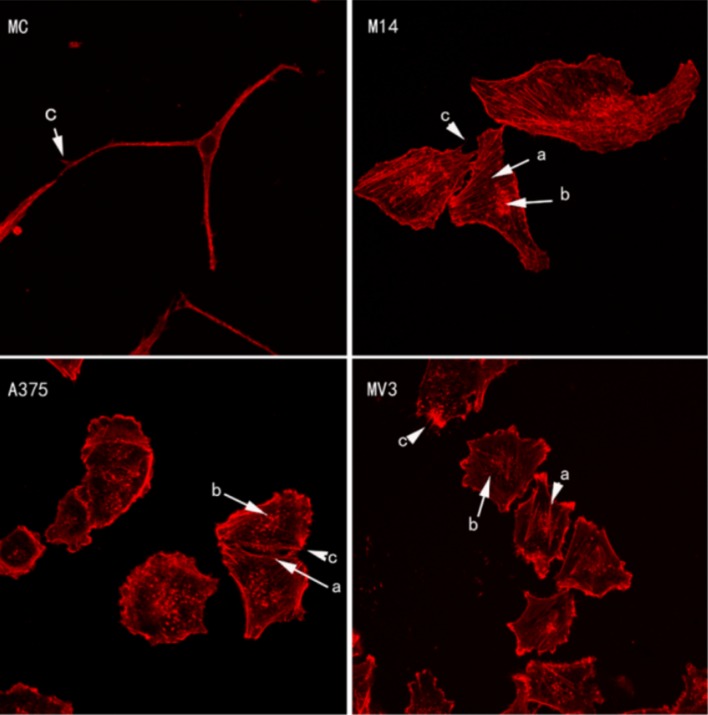
Cytoskeleton staining of 4 types of cells

As shown in Figure 2, MC was significantly different from 3 types of melanoma cells. MC did not have stress fiber (Figure 2, MC-b) and adhesion plaque (Figure 2, MC-a), while 3 types of melanoma cells had stress fiber (Figure 2, a) and adhesion plaque (Figure 2, b). Both MC and melanoma cells had thin and short filopodia (Figure 2, c). The stress fiber shape of MV3 was different from that of M14 and A375. MV3 had fewer but thicker stress fibers than the latter two.

### Transwell migration test

**Figure 3:**
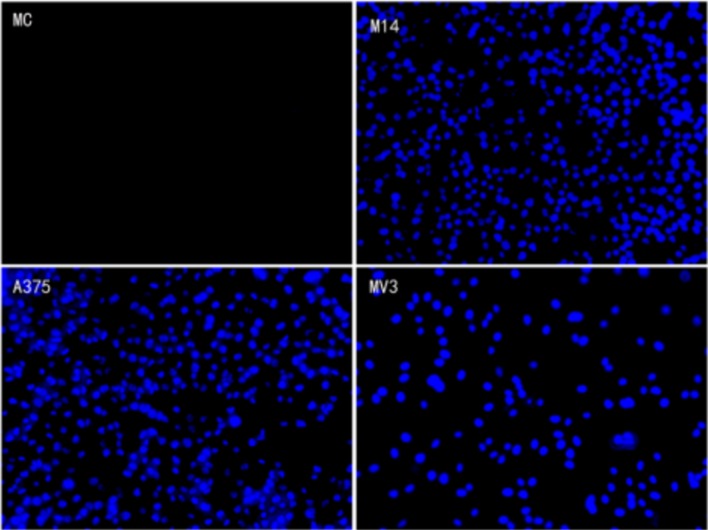
Comparison of invasion ability of 4 types of cells

**Figure 4:**
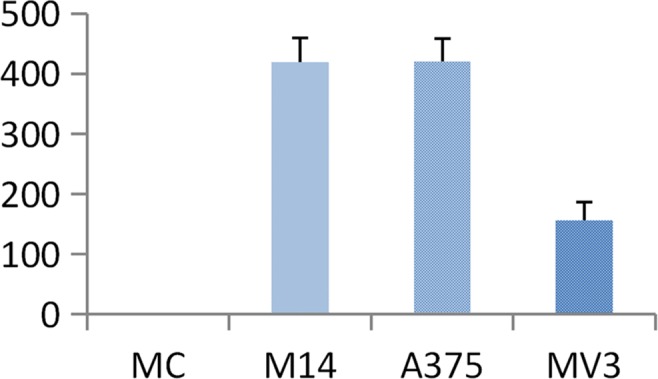
Transmembrane number of 4 types of cells in the migration test

As shown in Figure 3, the average transmembrane cells per field of group MC, M14, A375 and MV3 were (0±0), (419.33±40.01), (420.00±37.47) and (155.67±31.34), respectively. There was no significant difference between M14 group and A375 group (P>0.05). Statistically significant differences were found in the comparison of other groups (P<0.05) (Figure 4). Results indicated the followings: M14 and A375 had a similar high migration rate; the migration rate of MV3 was slightly low; MC did not have the ability of transmembrane migration.

### QPCR results

**Figure 5:**
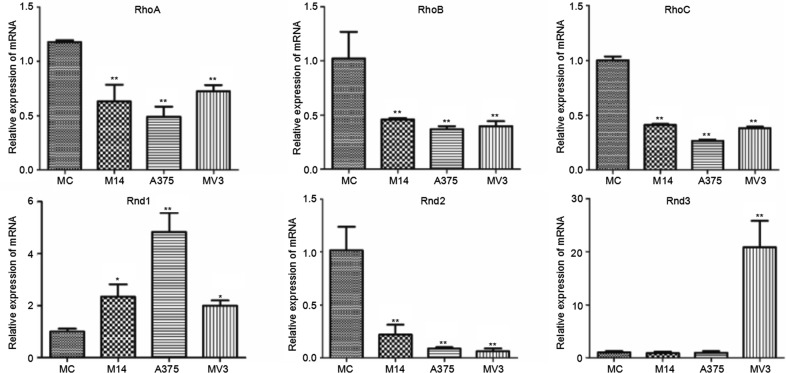
Differences in Rho subtribe and Rnd subtribe transcription among 4 types of cells

Rho subtribe includes RhoA, RhoB and RhoC. Rnd subtribe includes Rnd1, Rnd2 and Rnd3. As shown in Figure 5, compared with MC, the Rho subtribe transcription of melanoma cells reduced. Rnd1 had significantly higher expression in melanoma cells than in MC. The Rnd1 expression of A375 was higher than that of M14 and MV3. Rnd2 had lower expression in melanoma cells than in MC. Rnd3 had significantly higher expression in MV3. The Rnd3 expression differences between MC and M14, A375 were not statistically significant.

**Figure 6:**
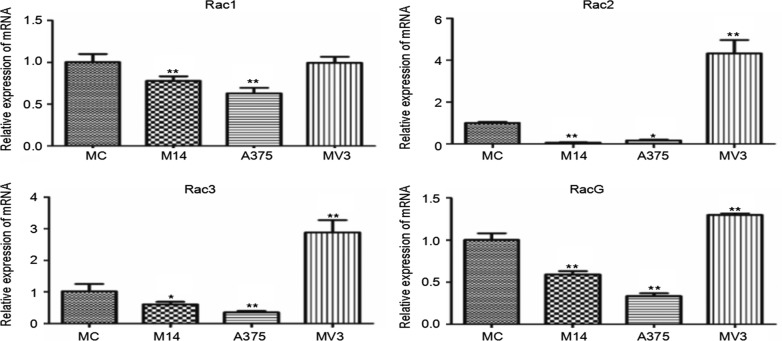
Differences in Rac subtribe transcription among 4 types of cells * Significant difference; ** Extremely significant difference.

Rac subtribe includes Rac1, Rac2, Rac3 and RhoG. As shown in Figure 6, there was no significant difference in the Rac1 transcription level between MC and MV3. MV3 had higher transcription levels of Rac2, Rac3 and RhoG than MC, M14 and A375. Compared with MC, M14 and A375 had lower transcription levels of Rac2, Rac3 and RhoG Figure 6.

**Figure 7:**
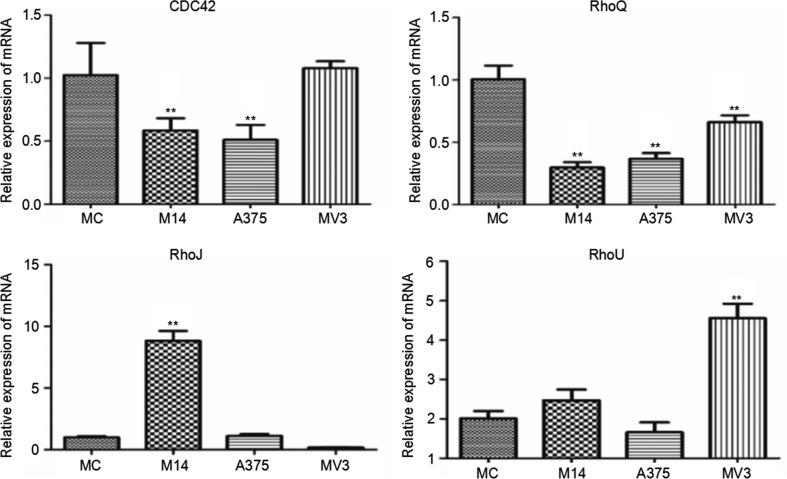
Differences in cdc42 subtribe transcription among 4 types of cells * Significant difference; ** Extremely significant difference.

Cdc42 subtribe is composed of cdc42, RhoQ, RhoU, RhoJ and RhoV. We found that RhoV was not expressed in all 4 types of cells. Compared with MC, M14 and A375 had a significantly lower cdc42 transcription level, but there was no significant difference between MC and MV3. The RhoQ transcription levels of 3 types of melanoma cells were lower than those of MC. The RhoJ transcription level of M14 cells was significantly higher compared to the other cells. The RhoU transcription level of MV3 cells was significantly higher compared to the other cells (Figure 7).

**Figure 8:**
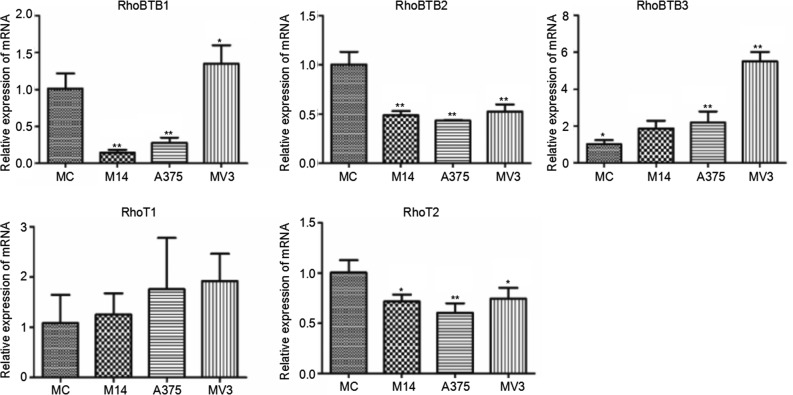
Differences in Rho BTB subtribe and Miro subtribe transcription among 4 types of cells * Significant difference; ** Extremely significant difference.

RhoBTB subtribe is composed of RhoBTB1, RhoBTB2 and RhoBTB3. Miro subtribe includes Miro-1(RhoT1) and Miro-2(RhoT2). The difference in RhoT1 transcription among 4 types of cells was not statistically significant. The transcription levels of RhoBTB2 and RhoT2 were significantly lower in melanoma cells than in MC. The RhoBTB1 transcription level of M14 and A375 was significantly lower than that of MC, but that of MV3 was higher than that of MC. The transcription level of RhoBTB3 was significantly higher in melanoma cells than that in MC (Figure 8).

**Figure 9:**
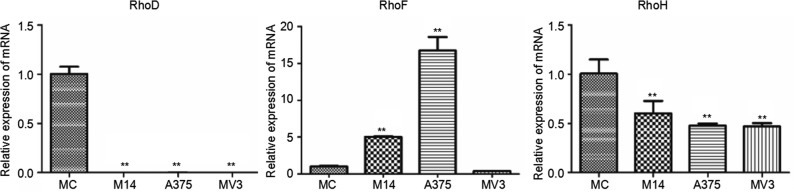
Differences in RhoD, RhoF and RhoH transcriptions among 4 types of cells * Significant difference; ** Extremely significant difference.

RhoD was rarely expressed in melanoma cells. The RhoD transcription level of melanoma cells was significantly lower than that of MC. A375 had significantly higher RhoF expression than the rest cells; M14 ranked the second in terms of RhoF expression; MV3 and MC had a similar RhoF transcription level which was significantly lower compared to the other two groups. The RhoH transcription level of melanoma cells was significantly lower than that of MC (Figure 9).

### WB results

**Figure 10:**
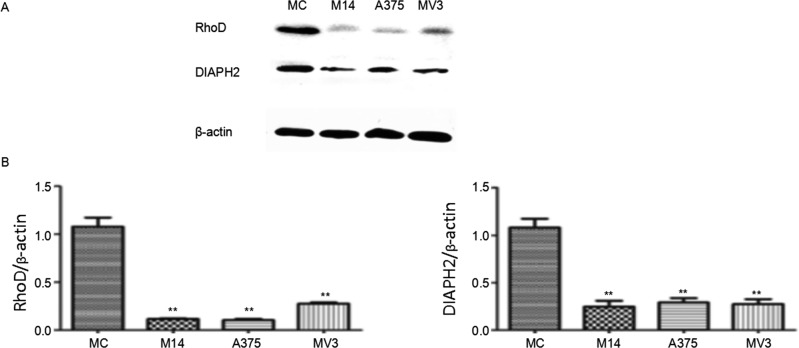
Expression of key proteins regulating cytoskeleton in melanoma cells and MC * Significant difference; ** Extremely significant difference.

WB results indicated that RhoD was the most special member. Literatures reveal that RhoD plays an important role in the migration and invasion of cells. As shown in Figure 10, the difference in RhoD protein was the same as the difference in the transcription level. RhoD was rarely expressed in melanoma cells, which was significantly lower than RhoD expression in MC. The expression of Diaph2, a downstream effector molecule of RhoD, was consistent with the expression of RhoD.

## DISCUSSION

There are only a few studies describing the influence of Rho GTPases family on the migration and invasion of melanoma through cytoskeleton. Our results indicated that Rho GTPases and filopodia, lamellipodia, stress fibers & adhesion plaque (the microfilament is the main constituent) showed significant and regular change in melanoma cells and MC.

Rho GTPases family play a key role in the recombination of actin cytoskeleton, and play an important regulatory role in the process of cell migration. After the activation, Rho GTPases regulate multiple cellular activities through signal transduction, such as cytoskeleton recombination, cell proliferation, cell polarization and cell chemotaxis.

Rho subtribe (including RhoA, RhoB and RhoC) is related to the formation of stress fibers and adhesion plaque, etc. [6]. QPCR results indicated that the expression of RhoA, RhoB and RhoC reduced in melanoma cells. Therefore, we conjecture that they contribute a little to the formation of stress fibers and adhesion plaque in MC and melanoma cells, but they may play other roles. A study shows that RhoA can regulate the expression of CD70--a TNF family. RhoA silencing can lead to the expression decline of CD70, while the overexpression of RhoA can induce the significant increase of CD70 expression [7], suggesting that RhoA may be related to the inhibition of melanoma. RhoB is a tumor-inhibiting factor as indicated by many studies [8]. The present study also found its reduced expression in tumor cells. Another report showed that overexpression of Rho subtribe can inhibit the expression of P21 (a tumor-inhibiting factor). These data reveal the precise regulation of cells. They also show that Rho subtribe may play a certain role in the inhibition of melanoma cells.

Rac subtribe (including Rac1, Rac2, Rac3 and RhoG) is related to the formation of lamellipodia and adhesion plaque. The expression levels of Rac2, Rac3 and RhoG were high in MV3, but extremely low in other cells, suggesting that these three had few effects on these cells. Although the Rac1 expression declined in M14 and A375 cells, the overall transcription level of Rac1 was very high. Although Rac1 can promote the formation of stress fibers and adhesion plaque, Rac1 overexpression can inhibit the migration and invasion of melanoma cells [9]. In addition to the above-mentioned function, Rac1 is also related to the stability of microtubules [10]. Quantitative data show that Rac1 is increased in MC, suggesting that its increase can increase the stability of microtubules. However, cell movement needs high degree of microtubule recombination. Therefore, we conjecture that the decrease of Rac1 expression promotes high degree of microtubule recombination in melanoma cells, and further promotes the migration and invasion ability of melanoma cells. Other members of Rac subtribe are highly expressed in MV3. On one hand, the migration and invasion ability is influenced by the stability of microtubules. On the other hand, their expressions promote the formation of lamellipodia in MV3 cells, which may be the reason why the lamellipodia of MV3 is significantly different from other groups.

Cdc42 subtribe includes cdc42, RhoQ, RhoJ, RhoV and RhoU. The expression of Cdc42 was very high in all 4 types of cells. Cdc42 is mainly related to the formation of thin and short filopodia in melanoma cells. RhoJ may be related to the formation of dorsal fold of cells. M14 had significantly higher RhoJ expression than other cells. Meanwhile, the dorsal fold of M14 was also significantly different from other cells. These indicate that RhoJ is closely related to the formation of M14 dorsal fold. Dorsal fold is also composed of microfilaments. Its function remains controversial and shall be further studied [11]. The RhoQ expression was lower in melanoma cells than in MC. RhoQ is related to the formation of lamellipodia. MC has extremely long and thin lamellipodia, which is significantly different from tumor cells. It means that RhoQ may be closely related to the formation of lamellipodia in MC. RhoU is related to the formation of stress fibers. MV3 cells had significantly higher RhoU than other cells. MV3 had thicker and lower density of stress fibers than other melanoma cells. Therefore, RhoU may play an important role in this process.

RhoD is closely related to stress fibers and adhesion plaque [12]. RhoD was highly expressed in MC, but was not expressed in melanoma cells. DIAPH2 is the downstream effector of RhoD. Western blot results indicated that the expression of RhoD and DIAPH2 proteins was consistent with the transcription level of RhoD. This indicates that RhoD can regulate the formation of stress fibers and adhesion plaque through DIAPH2, and influence the migration and invasion of melanoma cells. We also conducted relevant further study. After the overexpression of RhoD, we found the followings: the stress fiber of A375 became thin and weak; the adhesion plaque increased; the migration and invasion of A375 were inhibited. These results are the same as our expectation.

RhoF is closely related to stress fibers [13]. The same as Rnd1, RhoF expression in M14 and A375 was significantly higher than the rest two types of cells. The cytoskeleton staining indicated that the stress fiber of M14 and A375 cells was thinner and much more than that of the other two groups. A literature reports that Rnd1 is related to the loss of actin in stress fibers [14]. Therefore, we deduce that the stress fiber in M14 and A375 is the joint regulation result of RhoF and Rnd1. Rnd3 is related to the loss of stress fibers and adhesion plaque. In MV3, Rnd3 expression increased, and the quantity of stress fibers and adhesion plaque decreased significantly. Therefore, we conjecture that Rnd3 is closely related to the loss of stress fibers and adhesion plaque in MV3.

RhoH can inhibit the polymerization of microfilaments [15]. QPCR results indicated that RhoH expression was higher in MC than in M14, A375 and MV3. This suggests that RhoH inhibits the polymerization of microfilaments in MC, decreases the formation of stress fibers and adhesion plaque, reduces the power of cell migration, leads to the loss of anchorage force by cells, and further influences the cell migration.

In addition to the above-mentioned Rho GTPases members, some members of RhoBTB subtribe and Miro subtribe also displayed regular change as indicated in QPCR detection. However, whether they are related to the cytoskeleton change or not remains unclear at present. RhoBTB subtribe is related to the formation of tumor cells. RhoBTB1 and RhoBTB2 are highly homologous [16, 17]. QPCR results indicated that RhoBTB1 and RhoBTB2 jointly influenced the formation of M14 and A375, while only RhoBTB2 influenced the formation of MV3 cells. There is no report on the function of RhoBTB3 yet. We can see from QPCR results that RhoBTB3 also plays an important role in 3 types of melanoma cells, especially in MV3.

In addition, we did not perform experiment *in vivo*, which is a limitation of our study. In summary, cytoskeleton recombination is a must in cell movement, migration and invasion. The present study reveals that Rho GTPases influences the migration and invasion of melanoma cells through regulating filopodia, lamellipodia, stress fibers and adhesion plaque (the microfilament is the main constituent). The cytoskeleton and the migration movement are the accurate regulation results of different Rho GTPases members. The present study lays a foundation for later studies on the migration and invasion of melanoma.

## MATERIALS AND METHODS

### Cells and reagents

Primary human melanocytes (MC), human melanoma cell lines A375, M14 and MV3; phalloidin labeled by rhodamine; Transwell chamber; primary antibodies of RhoD, DIAPH2 and β-actin, secondary antibody of goat anti-rabbit labeled by HRP, BCA protein assay kit; reagents related to QPCR and WB.

### Morphology was observed by the Hoffman microscope

The morphology was observed when 4 types of cells were in good condition.

### Cytoskeleton was observed after the staining by phalloidin labeled by rhodamine

Four types of cells were digested and resuspended. Cells (4*10^5^/ml) were in inoculated to a 6-well plate with cover glass. The medium was added to achieve a total volume of 2ml. Cells were cultured for 24h. Cells were washed 3 times by PBS preheated at 37°C and fixed for 30min by 4% paraformaldehyde. Cells were then treated by 0.2% Triton x-100 for 10min. Cells were washed 3 times by PBS(5min/time), and sealed by 1ml 2% BSA for 2h. After phalloidin diluted by confining liquid was added, cells were incubated overnight at 4°C. Cells were washed 3 times by PBS (5min/time). After the addition of DAPI, cells were incubated for 5min. The cover glass was turned bottom upward on the glass slide. The nail polish was used to seal the cover glass. After airing, it was observed under the laser scanning confocal microscope (LSCM) with super-high resolution.

### Transwell migration test

Four types of cells were digested and resuspended. The 24-well plate was used. 500μl serum-free medium was added until just reaching the bottom of Transwell. Twenty thousand cells were mixed with 500μl medium containing 10% serum. Cells were inoculated to the upper Transwell chamber. Cells were cultured for 5h in an incubator until full adherence. The medium was removed. Serum-free medium was added to the upper Transwell chamber. The medium containing 10% serum was added to the lower chamber. The system was cultured overnight. 4% paraformaldehyde was added for 30-min fixation. Cells inside the upper chamber were removed by the cotton swab. DAPI staining for 10min was conducted. Cells were washed 6 times by pure water (5min/time). The quantity of nuclei was observed under the inverted fluorescence microscope and calculated.

### QPCR

Total RNA of cell samples was extracted. The cDNA was obtained through reverse transcription. Primers of members in Rho GTPases family were designed. Reaction system: 10ul 2 X SuperRealPreMix Plus; 0.8ul forward primer and 0.8ul reverse primer; 2ul cDNA template; 0.4ul 50 X ROX Reference Dye; 6ul Rnase-free H2O. Reaction conditions: Initialization at 95°C for 15min; denaturation at 95°C for 10s, annealing/extension at 60°C for 32s. Relative quantification was conducted by ΔΔCt method (Table 1).

**Table 1:** Rho GTPases family members and reference gene primer sequences

Gene	Forward primer	Reverse primer
RhoA	TAGCCAAGATGAAGCAGGAG	CACAAGACAAGGCACCCA
RhoB	CCGACATTGAGGTGGACG	GGGCACATTGGGACAGAA
RhoC	CAGTGCCTTTGGCTACCTT	CCTCCGACGCTTGTTCTT
Rac1	TCTGCCAATGTTATGGTAGATG	AGGACTCACAAGGGAAAAGC
Rac2	GTGATGGTGGACAGCAAGC	GGGAGAAGCAGATGAGGAAG
Rac3	GAGAATGTTCGTGCCAAGTG	TGGGTAGGTGATGGGTGC
RhoG	CCCAGCCTGACACCCTAC	GGCGAGCACTTCCTTGAC
Cdc42	GATTATGACAGATTACGACCGC	GAGTCCCAACAAGCAAGAAAG
RhoQ	ATGAGCTATGCCAACGACG	GGCTGGATTTACCACCGA
RhoJ	GTTACTGTGACTGTGGGAGGC	CCGTGTTGGGGTAGGAGA
RhoV	TCGTTCCCTTTGCTACCC	CGTTGACATCGTCCCTCAG
RhoU	AAAAGCCAGTGCCTGAAGA	TGTTGCTGAGTGTCCGAGTAT
RND1	CTCCTATGAGCAGGGTTGTG	GATGCCGTCCGAAAGATG
RND2	ATGTCCCCACCGTGTTTG	CAGTGTTTCTGGTCGGCTAA
RND3	AAGAATAGAGTTGAGCCTGTGG	AAGAGCATTTTGGTATTTGGAC
RhoD	AACAAGCTCCGAAGAAACG	GCAAAAGCCCTGGGTAAT
RhoF	AAGATCGTGATCGTGGGC	GAGGTGGGTGTTCTGGTAGG
RhoH	CAACTTGCCCTGTACCCC	CCTTGGCTCTGACATCCTG
RhoBTB1	GACGCTGACATGGACTACGA	CAACAACATCCCGAGAACG
RhoBTB2	GCCACCCTCACCCAGTAC	CTCCCATAAGCAAAGCGAC
RhoBTB3	AGGCAACCCACCATTACG	CATTTGAAACATCCCCAGAA
Miro-1	GGACGCTCACGACTTATTTAGA	AAGAACTCCACTTTTCCCACA
Miro-2	GAGATCCACAAGGCAAACG	CTCGGGAAACTGGCTCAT
GAPDH	AAATCCCATCACCATCTTCC	ATGACCCTTTTGGCTCCC

### Western blot

Total protein of cells was extracted. The total protein concentration was detected. Equivalent samples were subjected to SDS-PAGE gel electrophoresis and transfer. After the addition of primary antibodies with different concentrations, the protein was incubated at 4°C overnight. After washing, the incubation with secondary antibodies under room temperature for 1.5h was conducted. β-actin served as the internal reference.

### Statistical methods

Data were presented as mean ± standard deviation. One-way ANOVA was adopted for the statistical analysis. A P<0.05 indicated significant difference. The statistical software of SPSS19.0 was adopted.
